# Excision dynamics of *Vibrio *pathogenicity island-2 from *Vibrio cholerae: *role of a recombination directionality factor VefA

**DOI:** 10.1186/1471-2180-10-306

**Published:** 2010-11-30

**Authors:** Salvador Almagro-Moreno, Michael G Napolitano, E Fidelma Boyd

**Affiliations:** 1 Department of Biological Sciences, University of Delaware, Newark, DE 19716 USA; 2 Dartmouth Medical School, Dept. of Microbiology and Immunology, Vail Bldg. Rm 106, Hanover, NH 03755

## Abstract

**Background:**

*Vibrio *Pathogenicity Island-2 (VPI-2) is a 57 kb region present in choleragenic *V. cholerae *isolates that is required for growth on sialic acid as a sole carbon source. *V. cholerae *non-O1/O139 pathogenic strains also contain VPI-2, which in addition to sialic acid catabolism genes also encodes a type 3 secretion system in these strains. VPI-2 integrates into chromosome 1 at a tRNA-serine site and encodes an integrase *intV2 *(VC1758) that belongs to the tyrosine recombinase family. IntV2 is required for VPI-2 excision from chromosome 1, which occurs at very low levels, and formation of a non-replicative circular intermediate.

**Results:**

We determined the conditions and the factors that affect excision of VPI-2 in *V. cholerae *N16961. We demonstrate that excision from chromosome 1 is induced at low temperature and after sublethal UV-light irradiation treatment. In addition, after UV-light irradiation compared to untreated cells, cells showed increased expression of three genes, *intV2 *(VC1758), and two putative recombination directionality factors (RDFs), *vefA *(VC1785) and *vefB *(VC1809) encoded within VPI-2. We demonstrate that along with IntV2, the RDF VefA is essential for excision. We constructed a knockout mutant of *vefA *in *V. cholerae *N16961, and found that no excision of VPI-2 occurred, indicating that a functional *vefA *gene is required for excision. Deletion of the second RDF encoded by *vefB *did not result in a loss of excision. Among *Vibrio *species in the genome database, we identified 27 putative RDFs within regions that also encoded IntV2 homologues. Within each species the RDFs and their cognate IntV2 proteins were associated with different island regions suggesting that this pairing is widespread.

**Conclusions:**

We demonstrate that excision of VPI-2 is induced under some environmental stress conditions and we show for the first time that an RDF encoded within a pathogenicity island in *V. cholerae *is required for excision of the region.

## Background

**Figure 1:**
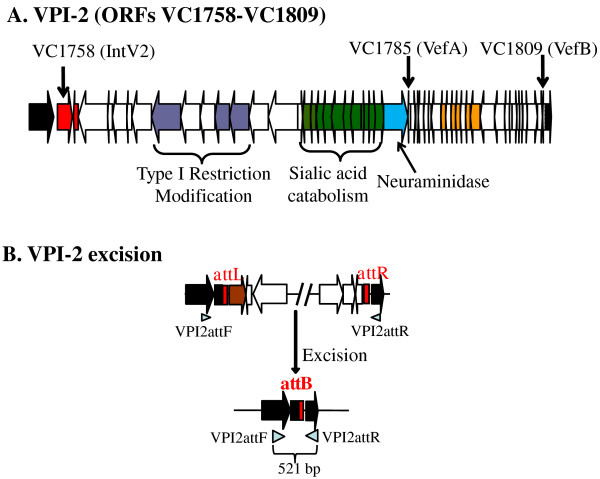
**Vibrio Pathogenicity Island-2 (VPI-2) ORFs and primers used in this study**. **A**. Schematic representation of VPI-2. Small black vertical arrows mark ORFs VC1758 (IntV2), VC1785 (VefA), or VC1809 (VefB). Block arrows represent ORFs and direction of transcription. Black arrows represent core genome ORFs (VC1757 and VC1810) present in all *V. cholerae *isolates; red arrow indicates an integrase, purple arrows indicate type 1 restriction modification ORFs, green arrows represent sialic acid catabolism, transport and regulator ORFs, blue arrow indicates sialidase/neuraminidase and yellow arrows represent transposase and Mu-phage like proteins. **B**. Schematic of VPI-2 excision mechanism and primer pair VPI2attF and VPI2attR used to detect the VPI-2 *attB *locus after excision of the entire region.

*Vibrio cholerae *is the etiological agent of the severe diarrheal disease cholera. Similar to many Gram-negative enteric pathogens, horizontal gene transfer and recombination plays a significant role in the evolution and emergence of new pathogenic strain of this species [1-12]. The main cause of the explosive rice water diarrhea characteristic of cholera is the cholera toxin (CT), an AB type enterotoxin, which is encoded within the ssDNA filamentous phage CTXɸ [13,14]. The B subunit of CT binds to the GM_1 _gangliosides, which are exposed when higher order gangliosides found in the intestinal mucus are cleaved by sialidase/neuraminidase (NanH). This protein is encoded within a 57 kb region named *Vibrio *Pathogenicity Island-2 (VPI-2) [15,16]. In addition to encoding sialidase, VPI-2 also encodes the sialic acid catabolism (SAC) gene cluster (Figure 1A) [16-19]. The SAC cluster was shown to be present only in pathogenic isolates of *V. cholerae *and enables the bacterium to grow on sialic acid as a sole carbon source [18,20]. Recently, we demonstrated that the ability to catabolize sialic acid gives *V. cholerae *a competitive advantage *in vivo *[19]. In non-O1/O139 pathogenic isolates, in addition to the SAC cluster are the genes required for a type 3 secretion system which is important for virulence [21-25]. The toxin co-regulated pilus (TCP), an essential intestinal colonization factor for *V. cholerae*, is encoded within the 40 kb *Vibrio *Pathogenicity Island-1 (VPI-1 or TCP Island) region [26,27].

VPI-1 and VPI-2 do not share any genes in common but do share some functional characteristics such as the ability to integrate into the chromosome, specifically at a tRNA site using an integrase belonging to the tyrosine recombinase family [16,18,23,26,28]. VPI-2 integrates into chromosome 1 at a tRNA-serine locus, whereas VPI-1 is located at the tmRNA locus. Both regions are flanked by direct repeats (DRs) named *attL *and *attR *[16,18,23,26,28]. These integrases, IntV1 (VC0847) and IntV2 (VC1758), are believed to mediate insertion into the host chromosome through site specific recombination between an attachment site *attP*, present in the pathogenicity island, and *attB*, present in the bacterial chromosome.

Pathogenicity islands have been shown to excise from their host genome in pathogenic *Escherichia coli *and *Yersinia *species [29-36]. In *E. coli *strain 536, a uropathogenic isolate, Hacker and colleagues have identified six PAIs, all of which encode a tyrosine recombinase integrase and are flanked by DRs [31,33,36-39]. They demonstrated that PAI-I, II, III and V can excise from the chromosome by site-specific recombination involving their respective DRs (*attL *and *attR*) [31,33]. The PAIs were shown to excise at different frequencies depending on the growth conditions [31,33]. Likewise, both VPI-1 and VPI-2 have been shown to excise from their host chromosome [23,28]. Rajanna and colleagues demonstrated that VPI-1 can excise from *V. cholerae *N16961 at very low rates [28]. They determined that the integrase IntV1 (VC0847) was not essential for excision since a transposase within the region appeared to compensate for an IntV1 knockout [28]. Recently, Murphy and Boyd demonstrated that VPI-2 from *V. cholerae *N16961 can excise from chromosome 1, which also occurred at very low frequency under optimal growth conditions [23]. Their study showed that IntV2 (VC1758) was essential for excision and the formation of a circular intermediate (CI) [23]. Pathogenicity islands from both *E. coli *and *V. cholerae *are non-self mobilizable, they do not encode any proteins such as those for phage structural proteins or conjugation systems needed for cell to cell mobility [23,28,31,33,36-39]. The mechanism of transfer for most pathogenicity islands remains to be elucidated but likely involves hitchhiking with plasmids, conjugative transposons, Integrative and Conjugative Elements (ICEs), or generalized transducing phages or uptake by transformation.

It is known that for some mobile and integrative genetic elements (MIGEs) the presence of a recombination directionality factor (RDF)/excisionase is required for excision [40,41]. For instance, Xis is required for the excision of the ICE SXT from *V. cholerae *[41], Hef from the High Pathogenicity Island of *Yersinia pestis *[32], and Rox from the *Shigella *Resistance Locus (SRL) of *Shigella flexneri *[42]. RDFs are small basic proteins that bind and bend DNA on the recombination sites *attL *and *attR *triggering excision by coordinating the assembly of the excisive intasome [43-45]. In addition, some RDFs have been found to inhibit reintegration of the CI by converting *attP *into a catalytically inactive structure and are thought to stabilize the appropriate positioning of the integrase within the excisive intasome [46-48]. To date, no RDFs have been identified in *E. coli *or *V. cholerae *pathogenicity islands.

Here, we report the environmental conditions that induce excision of VPI-2. We examined the VPI-2-encoded factors that are required for VPI-2 excision, determining that *V. cholerae *cells subjected to stress conditions showed an increase in the excision levels of VPI-2 compared to cell grown at optimal conditions. Bioinformatic analysis of the VPI-2 region identified two open reading frames (ORFs) VC1785 and VC1809 that show homology to previously described RDFs, which we named VefA and VefB. We examined the role of these genes in VPI-2 excision.

## Methods

### Bacterial strains and growth conditions

The strains and plasmids used in this study are listed in table 1. Bacteria were grown in lysogeny broth more commonly known as Luria-Bertani broth (LB), LB agar, or LB agar 10% sucrose without NaCl (LB-Suc) [49]. Strains harboring the pBAD33 expression vector were grown on LB supplemented with 0.02% W/V of L-Arabinose (LB-Ara). Bacteria were incubated overnight at 37°C with aeration unless otherwise indicated. When required, ampicillin (Amp, 100 μg/ml), streptomycin (Sm, 200 μg/ml), or chloramphenicol (Cm, 25 μg/ml) were added to the media.

**Table 1:** Bacterial strains and plasmids used in this study.

Strains/plasmids	Genotype and/or phenotype	Reference
*V. cholerae*		
N16961	O1 El Tor, VPI-2 +, Sm^R^	[57]
RAM-1	N16961, ΔVC1758, Sm^R^	[23]
SAM-1	RAM-1, pIntV2, Sm^R ^Cm^R^	This study
SAM-3	N16961, ΔVC1785, Sm^R^	This study
SAM-4	N16961, ΔVC1809, Sm^R^	This study
SAM-5	SAM3, pVefA, Sm^R ^Cm^R^	This study
SAM-11	N16961, pBAD33, Sm^R ^Cm^R^	This study
SAM-12	RAM-1, pBAD33, Sm^R ^Cm^R^	This study
SAM-13	SAM-3, pBAD33, Sm^R ^Cm^R^	This study
*Plasmids*		
pDS132	Suicide plasmid, Cm^R^, SacB	[59]
pBAD33	Expression plasmid, Ara, Cm^R^	[60]
pIntV2	*vc1758 *cloned into pBAD33	This study
pD1785	ΔVC1785 cloned into pDS132	This study
pD1809	ΔVC1809 cloned into pDS132	This study
pVefA	*vc1785 *cloned into pBAD33	This study

### Determination of VPI-2 excision rate

Excised circular VPI-2 DNA containing *attP *is expected to be a very rare event given the predicted low excision rate under normal conditions and the inability of VPI-2 to replicate after excision [23]. Therefore, we quantified the excision rates of VPI-2 by measuring the presence of *attB*, the locus present on the *V. cholerae *chromosome after VPI-2 excises (Figure 1B), in different strains under different conditions, and comparing it with the presence of *attB *in cultures of *V. cholerae *N16961 grown under standard optimal conditions: 12 hours in LB at 37°C with aeration. Using the O.D. values of 1 mL of a culture of *V. cholerae *N16961 grown for 12 hours in LB at 37°C with aeration as a reference, 750 μL to 4 mL were pelleted by centrifugation and genomic DNA was extracted using ABI PrepMan Ultra reagent from the test cultures. We took 50 μL from each DNA extraction and diluted each with 200 μL of sterile ddH_2_O. A 5 μL aliquot of DNA after dilution was used as template for Real-Time quantitative PCR (QPCR) reactions. The QPCR assay calculated the percentage of cells in a culture that contained an unoccupied VPI-2 *attB *site. We quantified *attB *sites present in cell grown under different growth conditions and normalized to the amount of *attB *present in N16961 grown for 12 hours at 37°C. The gene-specific primers were designed using Primer3 software according to the real-time PCR guidelines, and are listed in Table 2. The Applied Biosystems 7000 system was used for RT fluorescence detection of PCR products that resulted from binding of the dye SYBR Green to double stranded DNA and the results were examined with Applied Biosystems SDS software V 1.3. The reference gene *mdh *was assayed both separately and in the same reaction. To confirm that primer pairs only amplified target genes to assure accurate quantification of the results, non-template controls were included in each replicate. The *attB *and *mdh *PCR products were visually checked on agarose gels. The melting curves of PCR products were used to ensure the absence of primer dimers, contamination with genomic DNA and non-specific homologous sequences. PCR reactions were performed in 10 uL volumes containing 5 uL of 2X SYBR Green PCR Master Mix (Applied Biosystems), 900 nm of each primer, and 1 uL of DNA template. PCR cycling conditions were 30 sec at 95°C followed by 40 cycles of 15 sec at 95°C and 30 sec at 60°C. Serial doubling dilutions were used as templates for QPCR to generate standard curves for each PCR reaction by plotting relative DNA concentrations versus log (C_t_) value (C_t _is the PCR cycle at which fluorescence rises beyond background). The C_t _value for *mdh *was 15 cycles and for *attB *30 cycles. Every sample was assayed in triplicate and each experiment was performed using a minimum of three different samples. Differences in the *attB *ratio were extrapolated using the delta-delta Ct method as developed by Pfaffl [50].

**Table 2:** Oligonucleotide primers used in this study.

Oligo name	Sequence (5'-3'). Restriction site underscored	Target gene
*SOE PCR*		
VC1785A	GAGCTCAATGGTGCATCGGCATATTCT	*vefA *(A-D)
VC1785B	CAGCGATGATGGCGTGATTA	
VC1785C	TAATCACGCCATCATCGCTGGGATGT TCTCCTATGTCTTGT	
VC1785D	TCTAGACGCGCACATAACGCTGTTC	
VC1809A	CTGCAGTGAGAGCAAGGGAAGTGATCGT	*vefB*(A-D)
VC1809B	CGAGAGCTGGTTTACTTGTGTG	
VC1809C	CACACAAGTAAACCAGCTCTCGGTGCATGATTGTCAAGTCATGCA	
VC1809D	GAGCTCGATGGCTATGAATTAGCTCAGGA	
*Flanking*		
VC1785FF	GATGCTTCTATTACTCGGTT	*vefA *(F-R)
VC1785FR	TCACCGCTGCTGCGTTAA	
VC1809FF	GATTGATAGTAACAACACGCG	*vefB *(F-R)
VC1809FR	GTAATGCGCTATTGCTAAGTG	
VPI2attF	AGAGTGAAAGTCGCCAAAGC	*attB *(F-R)
VPI2attR	GGGTGCAATTTCGCATGTTGC	
*Complements*		
VC1758CF	GAGCTCGAGTCCTCATGCTCTAGCCAG	*intV2 *(F-R)
VC1758CR	TCTAGAGGCATGCTGGTGTGTTACTAC	
VC1785CF	GAGCTCGCTTTGAATATAGGTAAGGGACTG	*vefA *(F-R)
VC1785CR	TCTAGACTATAGTACATGACGCATGTATAATC	
*Real-Time PCR*		
VPI2attBQF	GATTCGGTGAGTTGTCCGAGT	attB (VPI-2) (F-R)
VPI2attBQR	GTGTTGGTGCAATGCTCAGTC	
VC1758QF	TGCATGATCTTATACTCACCG	*intV2 *(F-R)
VC1758QR	ACGTGCTCGCGGTTCATCTTC	
VC1785QF	CCGCACTAAGCCGTTCAGCAA	*vefA *(F-R)
VC1785QR	CCATCCACTCATCCACTTCGC	
VC1809QF	CTGAGAGGTGTGAATATGCCAG	*vefB *(F-R)
VC1809QR	GTGATCCGTTGAGCAATCCAC	
VC0432QF	TGTATGATATTGCGCCTGTCACAC	*mdh*(F-R)
VC0432QR	CCAGAACCACATCCGCACCTTC	

Underlined sequence represents restriction enzyme sites *SacI, XbaI and Pst1*.

### Bioinformatic analysis

BLAST search was performed using Xis (ABA87014), an RDF from *V.cholerae *SXT ICE element required for excision, AlpA, a well known RDF from *E. coli *(AAA18418) and the Hef protein (NP_405464) from *Y. pestis *pathogenicity island, as seeds on the genome sequence of *V. cholerae *N16961 [51]. DNA sequences from putative RDFs were downloaded from GenBank and the sequences were aligned using ClustalW [52]. Next, the protein sequences of characterized RDFs were used as seeds to perform BLASTN and BLASTP searches against *Vibrio *genomes sequences in the database [51]. The retrieved sequence must give an e-value below 10^-3^, relative to known RDFs.

### RNA extraction and Real-Time quantitative PCR (QPCR)

Total RNA from *V. cholerae *N16961 was extracted 12 hours post-inoculation in LB broth from one group treated with sub-lethal UV-irradiation and one group untreated as follows. The cells from 5 mLs 11 hours growth cultures were pelleted and resuspended in 5 mLs of PBS. A 100 uL aliquot was taken from each sample prior to treatment to calculate colony forming units (CFUs). Each 5 mL sample was placed in a plastic Petri dishes without a cover and one set of samples was irradiated with a sublethal dose of 25 J/m^2 ^of UV irradiation in a Fisher Scientific UV cross linker (FB-UVXL-1000) and the other 5 mL set of samples was left untreated as previously described by others [53]. The cells from both UV treated and untreated samples were recovered, pelleted, resuspended in 5 mLs of LB broth and grown for 1 hour at 37°C. A 100 uL aliquot was taken from each sample to calculate CFUs post treatment from both sets of samples. The CFU counts pre and post treatment were identical at ~9.75 × 10^9^/ml as expected. Every experiment was performed in triplicate. Total RNA was extracted from each culture using RNAprotect Bacteria reagent (Qiagen, Valencia, CA) and an RNeasy mini kit (Qiagen) according to the manufacturer's protocols. RNA purity and the presence of genomic DNA were assessed using an ND-1000 NanoDrop UV-Vis spectrophotometer (NanoDrop Technologies) giving values of *A*_260_/*A*_280 _> 2.0 and *A*_260_/*A*_230 _> 2.0 indicating of no protein and solvent contamination, respectively. In addition, 1 μg of each sample of RNA was run on a 1% agarose gel in 1× TBE buffer to examine quality of the samples. RNA was measured to calculate the volume of sample to be added to perform a reverse transcriptase (RT) reaction using SuperScript II Reverse Transcriptase and random hexamers following manufacturer's instructions (Invitrogen). The purity and quantity of cDNA was examined using an ND-1000 NanoDrop UV-Vis spectrophotometer as above. QPCR was performed using standard protocol using primer pairs for *vc1758, vc1785, vc1809 *and *vc0432 *(*intV2, vefA, vefB *and *mdh*, respectively) listed in Table 2 using SYBR green PCR Master Mix (Invitrogen) on an Applied Biosystems 7000 Real Time PCR System (Foster City, CA). To confirm that primer pairs only amplified target genes to assure accurate quantification of the results, non-template controls were included in each replicate. The *intV2, vefA, vefB *and *mdh *PCR products were visually checked on agarose gels. The melting curves of PCR products were used to ensure the absence of primer dimers, contamination with genomic DNA and non-specific homologous sequences. The data was analyzed using ABI PRISM 7000 SDS software (Applied Biosystems). Differences in the gene ratios were extrapolated using the delta-delta Ct method [50]. Every sample was assayed in triplicate and each experiment was performed using a minimum of three different samples.

### Construction of mutant strains

To construct the mutant strains, primers were designed to conduct Splice Overlap Extension (SOE) PCR followed by allelic exchange [54]. SOE PCR primers were designed to produce non-functioning constructs of the 204-bp *vefA *and the 228-bp *vefB *genes. The size of the regions removed from *vefA and vefB *is 169-bp and 191-bp, respectively and were constructed in *V*. *cholerae *strain N16961 to create mutant strains *V*. *cholerae *SAM-3 and SAM-4, respectively (Table 1). Primer pairs SOEVC1785A/SOEVC1785B and SOEVC1785C/SOEVC1785 D were used to amplify PCR products from VC1785 from *V. cholerae *strain N16961 (Table 2). The ligated product was amplified with primer pair SOEVC1785A and SOEVC1785 D, which was restricted with enzymes, *XbaI *and *SacI *and ligated with pDS132 (New England Biolabs) resulting in pΔ1785. pΔ1785 was transformed into *E. coli *strain DH5αλpir, plasmid purified and then transformed into *E. coli *β2155 cells. *E. coli *β2155 transformants were conjugated with N16961. *V. cholerae *cells were passaged in LB-suc to cure them of the integrated pΔ1785. PCR was used to screen for *V. cholerae *strains in which the wild type gene was replaced by the mutant gene, which was confirmed by sequencing. The Δ1785 strain was designated *V. cholerae *strain SAM-3. A knockout mutant of VC1809 was constructed in N16961 as described above using primer pairs listed in Table 2. Complementation of RAM-1 and SAM-3 mutant strains were generated by creating pIntV2 and pVefA, by cloning *intV2 *(VC1758) and *vefA *(VC1785), respectively into the SacI/XbaI sites of the expression plasmid pBAD33 (New England Biolabs) using standard cloning protocol (Table 1 and 2).

## Results and Discussion

### VPI-2 excision rates under different growth conditions

It was previously shown that the four pathogenicity islands identified in *V. cholerae *N16961 can excise from chromosome 1 and form circular intermediates (CI) [23,28]. The excision of VPI-1 and VPI-2 occurs at very low levels suggesting that excision is tightly controlled, although it may also suggest that the excision event is inefficient, possibly due to poor expression of the regulatory genes, an altered regulatory circuit, or mutations that might occur in these sequences as the region become evolutionarily integrated into the host chromosome [23,28]. First, we quantified the excision levels of VPI-2 in cultures of *V. cholerae *N16961 grown for 12 hours in LB at 37°C (standard conditions) by measuring the presence of *attB*, the locus present on the chromosome after VPI-2 excises (Figure 1), and comparing it with the housekeeping gene *mdh *using QPCR. We used *attB *as a surrogate for VPI-2 excision measurements since the copy number of *attP *in the CI is minuscule compared to *attB*, which replicates along with the rest of the chromosome unlike excised VPI-2. We compared the presence of *attB *with *mdh *since all cells encode one functional copy of the latter. PCR products of *attB *and *mdh *were visually checked on an agarose gel and their melting temperature analyzed to ensure we had the correct PCR products. The reference gene was assayed both separately and in the same reaction. Both primer pairs used were tested by comparing the results obtained using previously quantified cloned copies of *mdh *and *attB *and gave comparable results. We found that *attB *was present in 1 in every 1.6 (±0.2) × 10^6 ^*V. cholerae *cells under optimal growth conditions.

**Figure 2:**
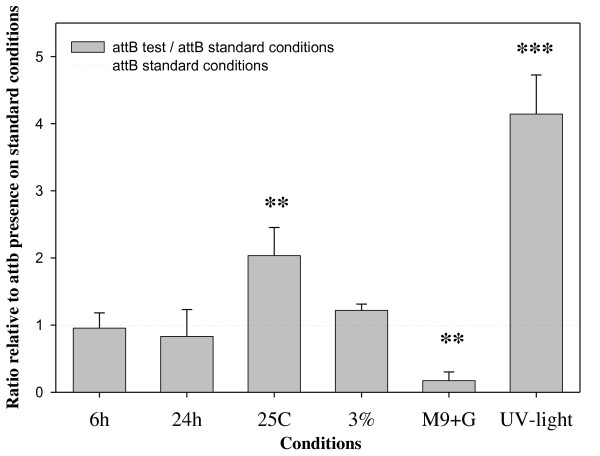
**Detection of VPI-2 excision by using real time quantitative PCR (QPCR) of *attB *levels in cell cultures grown under different conditions**. The X-axis specifies culture conditions: 6 h, incubation time of 6 h; 24 h, incubation time of 24 h; 25C, incubation temperature of 25°C; 3%, the LB broth contained 3% NaCl; M9+G, cell grown on minimal media supplemented with glucose; UV-light, bacterial cultures were UV-light irradiated. The Y-axis represent the ratio of the *attB *presence in the cultures tested compared with cultures grown on standard conditions 12 h at 37°C in LB. Unpaired *t*-test was used in order to infer statistical significance for the differences in VPI-2 excision. ***, p < 0.005. **, p < 0.05. Error bars indicate standard deviation. Each experiment was performed in triplicate a minimum of three times.

Next, we measured the presence of *attB *from *V. cholerae *cells cultured under different conditions compared with the presence of *attB *under our standard condition, growth for 12 hours at 37°C. We determined that incubation time does not affect the excision levels of VPI-2 indicating that excision does not occur in a growth phase dependent manner (Figure 2). However, *V*. *cholerae *cultures grown at 25°C showed a 2-fold increase in the presence of the *attB *site compared to cells grown at the optimum temperature 37°C (Figure 2). In addition, we found that nutrient limitation affected the excision level showing over a 5-fold decrease in the presence of *attB *when compared to the growth on LB at the same temperature (Figure 2). Furthermore, we found that sub-lethal UV-light irradiation of cell cultures compared to untreated cells, resulted in a significant increase in the level of excision of VPI-2, over 4-fold compared to untreated cells grown under the same conditions (Figure 2). Taken together, these data indicate that environmental factors can affect the induction of excision and circularization of VPI-2, which is probably the first step required for the horizontal transfer of the region. These results are consistent with what was previously shown for other mobile and integrative genetic elements as well as PAIs from *E. coli*, where excision occurs upon exposure to stress conditions such as sub-lethal UV-light irradiation [53,55,56].

### VPI-2 encodes two novel recombination directionality factors

**Figure 3:**
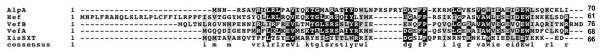
**Alignments of VPI-2 RDFs VefA and VefB with other known RDFs: AlpA (AAA18418), Hef (NP_405464), Xis (ABA87014)**. For both alignments black shading highlights residues shared by all the sequences and grey shading highlights residues shared by all but one sequences. The numbers on the right indicate the number of amino acids of the predicted protein.

Both the high pathogenicity island HPI from *Y. pestis *and ICE SXT from *V. cholerae *encode small accessory proteins called recombination directionality factors (RDFs) or excisionases (Xis) that are required for efficient excision of these elements [29,41]. In order to identify candidate RDFs within VPI-2 from *V. cholerae *N16961, we performed BLAST and PSI-BLAST searches on the *V. cholerae *N16961 genome using RDFs, the *V. cholerae *Xis protein (ABA87014) from SXT, the *Y. pestis *Hef protein (NP_405464) from HPI and *E. coli *K12 AlpA protein (AAA18418) from λ phage as queries [57]. The most significant BLAST result in these searches was ORF VC0497, which is annotated as a transcriptional regulator, and is encoded within Vibrio Seventh Pandemic island-II (VSP-II). VSP-II also encodes a tyrosine recombinase integrase at ORF VC0516 (IntV3) [58]. ORFs VC1785 and VC1809 encoded within VPI-2 were the second and third most significant hits retrieved from these BLAST searches, which we termed VefA (for *Vibrio *excision factor A) and VefB, respectively (Figure 3). The VefA and VefB proteins share 46% amino acid identity/72% similarity. VefA shares 37% amino acid identities with AlpA, 46% identity with Hef and 29% with Xis from the *V. cholerae *SXT element as was previously shown [53] (Figure 3). The *vefB *gene is located at the 3' end of VPI-2 at ORF VC1809 marking the end of the island, and *vefA *(VC1785) is adjacent to neuraminidase gene, *nanH *(VC1784) in the middle of the island (Figure 1A).

**Figure 4:**
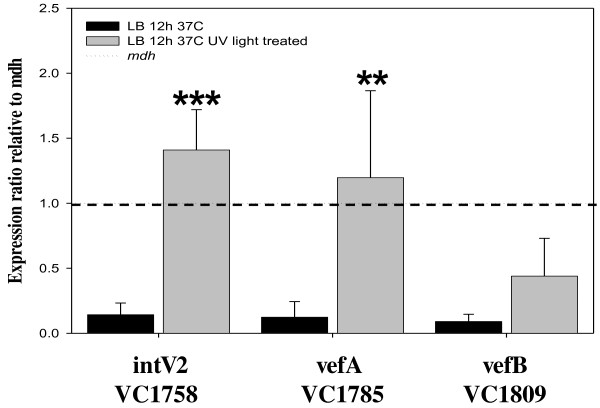
**Expression of *intV2 *(VC1758), *vefA*, and *vefB *from cultures grown in standard (black bars) or UV-light irradiated cultures (grey bars)**. The Y-axis represents the expression ratio of the genes relative to the expression of *mdh*. Unpaired *t*-test was used in order to infer statistical significance for the differences in gene expression between cultures of *V. cholerae *N16961 with or without UV-light treatment. **, p < 0.05; ***, p < 0.005. Error bars indicate standard deviation. Each experiment was performed in triplicate a minimum of three times.

As shown in Figure 2, UV-light irradiation increased excision of VPI-2 over 4-fold. In order to investigate this further, we determined the effect of UV-light irradiation on the expression of *intV2, vefA *and *vefB *in *V. cholerae *N16961 (Figure 4). We examined transcript levels of *intV2, vefA and vefB *in cells grown for 12 h in LB and in cells grown for 12 h in LB followed UV-light irradiation treatment. We found that all three genes showed negligible levels of transcription under standard optimum growth conditions but after UV-light treatment both *intV2 *and *vefA *show a 10-fold and *vefB *a 5-fold increase in expression levels (Figure 4). These results indicate that UV-light induces expression of factors potentially involved in VPI-2 excision.

### IntV2 and VefA are essential for the excision of VPI-2

**Figure 5:**
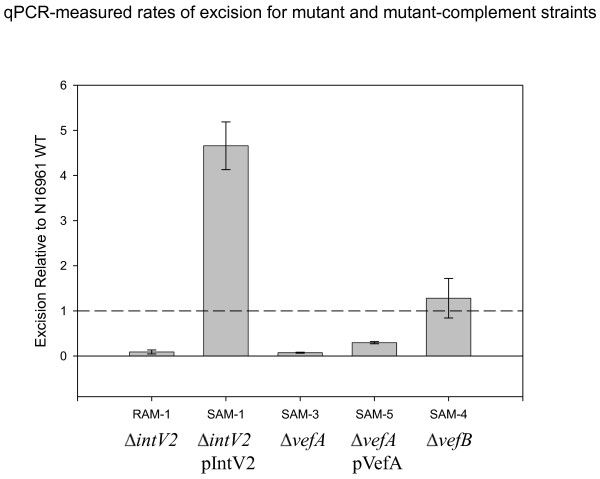
**Excision levels of VPI-2 in mutant strains and strains complemented with *intV2 *(VC1758), and *vefA *(VC1785)**. Excision levels of Δ*intV2 *mutant (RAM-1), Δ*intV2 *mutant complemented (SAM-1), Δ*vefA *mutant (SAM-3), Δ*vefA *mutant complemented (SAM-5), and Δ*vefB *mutant (SAM-4). Unpaired *t*-test was used in order to infer statistical significance for the differences in VPI-2 excision between *V. cholerae *N16961 and test strains. **, p < 0.05; ***, p < 0.005. Error bars indicate standard deviation. Each experiment was performed in triplicate a minimum of three times.

To determine in more detail the role of *intV2, vefA *and *vefB *in VPI-2 excision, we created deletion mutations in each gene and measured excision levels of VPI-2 by determining *attB *levels in cells. In *V. cholerae *RAM-1, an *intV2 *mutant, we did not detect any VPI-2 *attB *products, demonstrating that *intV2 *is essential for excision as was previously shown (Figure 5) [23]. We complemented RAM-1 with a functional copy of *intV2 *by transforming *V. cholerae *RAM-1 with pIntV2 creating strain SAM-1. In our SAM-1 strain, we found that excision of VPI-2 was restored in addition, *attB *levels were approximately four-fold higher than wild-type levels which is represented by the dotted broken horizontal line in Figure 5. These data demonstrate that over expressing *intV2 *ectopically induces excision of VPI-2. In our control experiments, transformation of either wild-type N16961 or RAM-1 with pBAD33 alone (strains SAM-11 and SAM-12 respectively) did not affect *attB *levels (data not shown).

Next, we determined whether one, both, or neither of the putative RDFs uncovered by our bioinformatic analysis are required for VPI-2 excision. To do this, we constructed in-frame deletion mutations in each gene to create mutant strain SAM-3 (Δ*vefA*) and SAM-4 (Δ*vefB)*. The two mutant strains and the wild-type N16961 were each inoculated into LB and all three strains grew similarly indicating that the mutant constructs did not have any general growth defect (data not shown). We determined the *attB *levels using QPCR in strain SAM-3 compared to the wild-type strain grown under the same conditions. We found that no VPI-2 excision occurs in SAM-3 cells when compared with the wild type, indicating that a functional copy of *vefA *is essential for efficient excision of VPI-2 (Figure 5). We complemented SAM-3 with a functional copy of *vefA *(SAM-5) and measured *attB *levels in these cells with the wild type levels both under standard conditions, to find that some excision occurred, but it was less than in wild-type cells (Figure 5). In our *vefB *mutant strain (SAM-4), we found no difference in VPI-2 excision levels compared to wild-type grown under the same conditions, which demonstrates that *vefB *is not essential for excision (Figure 5). From these data it appears that *vefA *is the cognate RDF for VPI-2 excision. In our control experiments, transformation of SAM-3 with pBAD33 alone (resulting in strain SAM-13) did not affect *attB *levels (data not shown).

### *Vibrio *species island-encoded integrases with corresponding RDFs

Given that our initial search for RDFs within one *V. cholerae *genome (strain N16961) yielded three putative RDFs (VC0497, VC1785, and VC1809), we decided to investigate further the occurrence of RDFs among *Vibrio *species whose genome sequence is available in the database. We performed BLAST searches against the 20 *Vibrio *species in the genome database, and we uncovered a total of 27 putative RDFs (Table 3). Next, we identified putative integrases within the genomes of the RDF homologues using BLAST search analysis by using IntV2 as a seed. For each of the RDFs identified among the 27 genomes encompassing 10 different *Vibrio species (V. cholerae, V. coralliilyticus, V. furnissii, V. harveyi, V. parahaemolyticus, V. splendidus, V. vulnificus, Vibrio *sp. Ex25, RC341, and MED222), we identified a corresponding integrase with greater than 40% amino acid identities to IntV2 (VC1758) (Table 3). We examined the gene context of each RDF and integrase within each of the 20 strains to determine whether the RDF and integrase were located on the same region within a strain. From these analyses, we found that each of the 27 RDFs has a corresponding integrase within approximately 100 kb of each other (Table 3). It should be noted that from table 3, only three of the strains have been annotated completely and for many of the strains examined their ORF annotation numbering is not consecutive. Within *V. cholerae*, integrases and RDFs located in the same region of the genome in different strains had the same gene content indicating the same island is present in different strains. Among the different species, however, integrases and RDFs associated with the same insertion site did not have the same gene content indicating a novel island region in the different species (data not shown).

**Table 3:** Locus tags for integrases and corresponding RDFs identified in this study.

	Integrases	RDFs
**Species Strain**	**Locus tag**	**Locus tag**

*Vibrio cholerae *N16961*	VC1758	VC1785/VC1809
*Vibrio cholerae *TM 11079-80	VIF_001175	VIF_000799
*Vibrio cholerae *TMA21	VCB_002798	VCB_002857
*Vibrio cholerae *12129(1)	VCG_002315	VCG_002259
*Vibrio cholerae *V51	VCV51_1204	VCV51_0550
*Vibrio cholerae *1587	A55_1986	A55_2025
*Vibrio cholerae *CT 5369-93	VIH_002346	VIH_002364
*Vibrio cholerae *RC385	VCRC385_0574	VCRC385_3603
*Vibrio cholerae *TMA 21	VCB_000212	VCB_000197
*Vibrio cholerae *MZO-3	A51_B0496	A51_B0476
*Vibrio cholerae *12129(1)	VCG_003155	VCG_003160
*Vibrio cholerae *N16961*	VC0516	VC0497
*Vibrio cholerae *MZO-3	A51_B0965	A51_B0948
*Vibrio vulnificus *YJO16*	VV2262	VV2261
*Vibrio vulnificus *YJ016*	VV0817	VV0810
*Vibrio vulnificus *YJO16*	VV0560	VV0515
*Vibrio furnissii *CIP 102972	VFA_001916	VFA_001914
*Vibrio furnissii *CIP 102972	VFA_000464	VFA_000468
*Vibrio coralliilyticus *ATCC BAA-450	VIC_001980	VIC_001987
*Vibrio *sp. Ex25	VEA_004301	VEA_004310
*Vibrio *sp. RC341	VCJ_000330	VCJ_000314
*Vibrio *sp. MED222	MED222_15534	MED222_15529
*Vibrio splendidus *12B01	V12B01_04993	V12B01_05053
*Vibrio parahaemolyticus *AQ3810	A79_5467	A79_5463
*Vibrio parahaemolyticus *K5030	VparK_010100010115	VparK_010100010135
*Vibrio parahaemolyticus *AQ3810	A79_2546	A79_2541
*Vibrio harveyi *HY01	A1Q_2023	A1Q_2003

* indicates a genome that is completely annotated

From our analysis, no RDF was identified within the VPI-1 or the VSP-I regions in N16961 or within homologous regions in the other 27 sequenced strains of *V. cholerae *in the database. Both the VPI-1 and VSP-I regions have been shown to excise from their chromosome location, and VPI-1 encodes a tyrosine recombinase with homology to IntV2, thus they may therefore use an alternative mechanism of excision or perhaps co-opt an RDF from another region on the genome. Overall our data indicates that the presence of both an integrase and a cognate RDF pairing is a relatively conserved feature but not an essential one.

## Conclusions

In this study, we analyzed the excision dynamics of VPI-2 encoded within *V. cholerae *N16961. Our results indicate that excision is controlled by at least two conserved factors within the island, an integrase encoded by *intV2 *and an RDF encoded by *vefA*, whose expression is induced by environmental stimuli similar to other MIGEs such as prophages, ICEs and integrons. We identified two putative RDFs and found that of the two we identified, only one VefA is essential for the efficient excision of VPI-2. We determined the occurrence of RDFs among the genomes of sequenced *Vibrio *species and found 27 putative RDFs that also had a homologue of IntV2 associated with it, which suggests that requirement for both an RDF and a corresponding integrase is a relatively common feature.

## Authors' contributions

EFB designed the research; SA-M and MGN performed the research; SA-M, MGN and EFB analyzed data; SA-M, MGN and EFB wrote the paper.
